# Effect of Solvent Extraction Parameters on the Recovery of Oil From Spent Coffee Grounds for Biofuel Production

**DOI:** 10.1007/s12649-017-0061-4

**Published:** 2017-08-31

**Authors:** Ioannis Efthymiopoulos, Paul Hellier, Nicos Ladommatos, Arthur Kay, Ben Mills-Lamptey

**Affiliations:** 1 Department of Mechanical Engineering, University College London, Torrington Place, London, WC1E 7JE UK; 2 Bio-bean Ltd., 6-8 Cole St., London, SE1 4YH UK

**Keywords:** Spent coffee grounds, Solvent extraction, Biodiesel, Coffee lipids, Fatty acid profile, Free fatty acids

## Abstract

Spent coffee grounds (SCG) are a potentially valuable source of lipids for sustainable production of biofuels. However, there are several feedstock properties and solvent extraction parameters that can impact on the oil yield and quality, potentially reducing the possible environmental benefits of deriving oils from this waste stream. This study presents results of laboratory and pilot plant scale experimental investigations into lipid recovery from spent coffee, determining the effects of solvent extraction variables including duration, SCG-to-solvent ratio and SCG residual moisture. SCG samples from both the industrial production of instant coffee and retail coffee shops were characterized in terms of moisture content, particle size distribution and oil content to identify the impact of these variables on the efficiency of lipid recovery by solvent extraction. The dry weight oil content of the instant SCG samples ranged from 24.2 to 30.4% w/w, while the retail SCG samples contained considerably lower amounts of lipids with their oil content ranging between 13.4 and 14.8% w/w. The highest oil yields were found at an extraction duration of 8 h, while a moisture content of ~2% w/w led to increased yields relative to completely dry samples. A pattern of increasing acidity with decreasing extraction duration was observed, suggesting preferential extraction of free fatty acids (FFA), with the fatty acid (FA) profile of the oil found to be similar to lipids commonly utilized for biofuel production.

## Introduction

Coffee beans are one of the most commonly used agricultural products used in the beverage industry, with the annual worldwide production of coffee beans estimated at 8 million tons [1]. The bulk of the coffee produced is harvested, roasted and sold for human consumption, and spent coffee grounds (SCGs), the solid residues that remain after the brewing process, are a significant waste material stream with the instant coffee industry alone generating 6 million tons of SCG worldwide per year [2]. Utilization of SCGs is therefore a pressing issue as they are at present mostly discarded as waste to landfill, causing pollution issues attributable to the presence of caffeine, polyphenols and tannins [3, 4].

Several approaches to the valorization of SCGs have been made, including utilization as fertilizers or wood powder, as a source of antioxidant and polysaccharide material or as an absorbent for removing cationic dyes in wastewater treatments, however these strategies have not been routinely implemented at an industrial scale [3, 5, 6]. Meanwhile, there has recently been increased interest in using SCGs as a biodiesel feedstock [7].

However, a potential disadvantage of the use of SCGs as a biodiesel feedstock is their relatively high moisture content, which usually has to be removed prior to oil extraction. SCGs normally contain moisture varying from between 50 and 85% w/w depending on the brewing process used [8, 9], though have also been reported as containing lower levels of moisture of between 18–45% w/w by Deligiannis et al. [10]. In general, SCGs from the instant coffee industry retain higher water levels than SCGs generated from coffee bars [9]. The water is present either as unbound excess moisture arising from the coffee brewing process, or bound moisture entrapped within the microstructure of the solid particles, levels of which vary with the origin and type of bean [8]. Gomez-de la Cruz et al. investigated the effect of SCG sample thickness, drying duration and temperature on the efficiency of the drying process and found that drying time decreases with temperature increase and sample thickness decrease [9].

The lipid content of SCGs on a dry weight basis has been found to range between 7 and 27.8% w/w according to previous studies [6–8, 10–17] and is significant relative to other major biodiesel feedstocks such as rapeseed oil (37–50%), soybean oil (20%) and palm oil (20%) [18]. Coffee oil consists primarily of triglycerides, diglycerides, monoglycerides and FFAs that combined account for 85–90% of the total lipid content and are principally composed of linoleic, palmitic, oleic and stearic acids in decreasing order of magnitude and in proportions not unlike those found in other common edible vegetable oils [6, 11, 13, 16, 19]. The FA composition of the extracted oil is significant as it affects the properties of the derived FAMEs, while the presence of FFAs is a major factor affecting the potential for efficient further processing into biodiesel as high FFA levels increase susceptibility to oxidation, speed up degradation and inhibit alkaline catalyzed transesterification [20–22].

The remaining component of coffee oil consists of a relatively large proportion of unsaponifiable compounds including diterpenes, sterols, tocopherols, phosphatides and waxes [23, 24]. Furthermore, coffee oil contains antioxidants which increase the stability of the oil and prevent decomposition [8]. In particular, the nitrogenous brown-colored compounds of coffee (Maillard reaction products) exhibit antioxidant capacity and inhibit lipid peroxidation [14, 25], while the diterpenes kahweol and cafestol and phenolic compounds contained in coffee oil are also known for their antioxidant activity [14].

The variation found in SCG lipid content (between 7 and 27.8% w/w) can in part be attributed to factors such as the different blends of coffee varieties (Arabica ~15%, Robusta ~10%), the origin of the coffee beans (cultivation climate, time of picking) and the upstream processing (wet or dry processing and roasting) [8, 11, 23, 26]. In addition, it has been seen that the oil yield obtained from SCGs depends on the extraction method, moisture content, particle size, type and volume of solvent and extraction time [8, 15, 17]. Soxhlet solvent extraction has been the baseline method used in most of the previous studies investigating lipid extraction from SCGs, with *n*-hexane regarded as the most effective solvent in studies which have considered a range of solvents [7, 8, 12, 27]. Table 1 presents the oil yields achieved by Soxhlet extraction with hexane reported previously, including the coffee to solvent ratio and extraction duration used where this information was available. The SCGs used in all the studies presented in Table 1 were provided from coffee shops local to the investigators, and would likely have contained differing absolute oil contents, complicating the direct comparison of these results. In particular, it is therefore difficult to ascertain an effect of Soxhlet extraction duration on oil yield.

**Table 1:** Soxhlet extraction oil yields on a dry weight basis reported in other studies when hexane is the solvent used

Study	Oil yield on dry weight basis (%) w/w	Duration of extraction (h)	Coffee to solvent ratio (w/v)
Kondamudi et al. [7]	13.4	1	1:3
Al-Hamamre et al. [8]	11.2–15.28	0.25–0.5	1:4.2
Couto et al. [12]	18.3	**–**	**–**
Ahangari and Sargolzaei [13]	16.7	6	1:15
Abdullah and Bulent Koc [16]	13	8	1:15
Caetano et al. [17]	16	2.5–9.5	1:20
Deligiannis et al. [10]	10–15	**–**	**–**

The effect of Soxhlet extraction duration has however been partly addressed by Al-Hamamre et al. for short durations of between 15 and 30 min, but without revealing a clear correlation between oil yield and extraction time [8]. Utilizing a non-recirculating solvent extraction method, Pichai and Krit, further investigated the effect of extraction duration and coffee to solvent ratio by immersing SCGs in hexane at coffee to solvent mass ratios ranging from 1:5 to 1:25 for durations up to 40 min at a temperature of 30 °C achieving the highest oil yield (14.68% w/w) at a ratio of 1:22.5 after 30 min of extraction [15]. A correlation between decreasing coffee to solvent ratio and improved oil yield was found, suggesting increased solvent quantities to enhance oil diffusion, while prolonged duration of extraction only slightly increased oil yield [15]. However, it should be noted that this study cannot directly be related to Soxhlet extraction, which includes numerous cycles of evaporation and condensation of the solvent and thus avoids saturation of the solvent with extracted oil [28].

Picard et al., who investigated the extraction of lipids from fresh roasted Robusta grounds, found that with increasing extraction time from 6 to 8 h the oil yield increased from 11.4 to 11.6% w/w, and then slightly decreased at longer durations of 10 and 12 h to 11.0 and 10.9% w/w respectively [29]. The effect of extraction duration on the oil yield obtained from other oilseeds (soybean, sunflower, cotton) by Soxhlet solvent extraction has been previously investigated with durations between 3.5 and 5 h found to be optimal as shorter durations reduced oil yields, while further increase in extraction duration did not further increased yields [30, 31]. Another study examining the Soxhlet extraction of oil from jatropha seeds concluded that most of the oil is extracted after 6 h, although a slightly higher oil yield was achieved after 8 h [32].

The effect of seed to solvent (hexane) ratio on extraction efficiency of oil from soybeans was investigated by Bulent Koc et al. through ultrasound assisted Soxhlet extraction, with a correlation between increasing seed to solvent ratio from 1:10 to 1:2 and increasing oil yield found [33]. Similar observations have been made when extracting lipids from soybean, sunflower and cottonseed at seed to solvent ratios of 1:1, 1:5 and 1:10 [31]. However, Sayyar et al. found that the efficiency of the lipid extraction from jatropha seeds increased when the seed to solvent ratio was decreased from 1:4 to 1:6 and a marginal increase in oil yield was achieved with a ratio of 1:7 [32]. It was theorized that this could be attributed to an increase in oil yield with a low solid to solvent ratio up to a limit due to the decrease in the concentration gradient between solid and liquid phase which favors mass transfer, in agreement with Pichai and Krit when investigating lipid extraction from SCGs [15, 32].

The effect of SCG moisture content and particle size on the efficiency of solvent extraction of lipids has not been previously addressed. However, in studies examining the extraction of lipids from other oilseeds, an intermediate moisture content of 9–11% w/w results in peak oil yields, while higher levels of moisture were found to interfere with the solvent penetration and oil diffusion as hexane is highly insoluble in water [30, 31, 34, 35]. Lower feedstock moisture contents have been observed to result in lower yields due to the reduced solubility of phosphatides in the absence of water [35].

With regards to the impact of particle size, previous studies have highlighted the need for size reduction by grinding prior to solvent extraction in order to increase surface area of oilseeds (e.g. soybean) and facilitate oil removal [36, 37]. These have most commonly investigated the effect of seed flake thickness that has been found to linearly correlate with average particle size, with a particle size of 0.22 mm corresponding to a flake thickness of 0.18 mm [30]. Seed flakes of thicknesses between 0.2 and 0.3 mm were reported to result in higher oil extraction rates, while a decrease in flake thickness from 5 to 0.5 mm resulted in yield increases in the case of oil extraction from soybean, cottonseed and sunflower [30, 31, 35–37]. Generally small and thin flakes offer good solvent permeability and oil diffusion but poor percolation of solvent, while large thick seeds flakes possess inverse properties due to surface area decrease [35]. It has therefore been suggested that a balance between these two limiting conditions produces optimum oil yields [30, 31, 35–37].

Sayyar et al. investigated the extraction of lipids from jatropha seeds with various particles sizes and found that an intermediate size particle (0.5–0.75 mm) resulted in the highest oil yields, with lower oil recoveries being reported in the case of particles larger than this relative to those <0.5 mm in diameter [32]. The reduction in oil yield observed when jatropha seed particles smaller than 0.5 mm were used, with only 40% of oil being extracted relative to the size range of 0.5–0.75 mm, was attributed to agglomeration of fine particles that reduces the surface area available for solvent flow [32]. On the contrary, Folstar et al., used sieves of 0.12 mm up to 0.85 mm to size green Arabica coffee grounds and observed a slight decrease in the oil yield as the particle size increased [38].

In this work, several SCGs samples from both industry and retail sources were characterized in terms of oil and moisture content and particle diameter distribution. Soxhlet extraction was then used to determine the influence of these SCGs physical properties, and the effects of extraction duration and coffee to solvent ratio on the yield of oil obtainable from the grounds. Most of the factors considered such as moisture content, coffee to solvent ratio and particle size of SCGs have not been investigated in depth before, while the effect of the duration of extraction has been only partially addressed [8]. Experimental results are also compared with oil yields obtained by a large scale pilot plant, offering for the first time a useful insight into the potential industrialization of the process. The FA profile and FFA content of selected oil samples was also determined for the purpose of identifying differences in the composition of oils extracted that may occur due to variation of solvent extraction parameters.

## Materials and Experimental Methods

The majority of the samples used in the experimental part of this study were provided by Bio-bean Ltd., while some further experiments were conducted with fresh (pre-brewing) and SCG samples from a local coffee shop. Information regarding the origin and upstream processing of most of the samples used was not available, however, 3 of the SCG samples were derived from instant coffee production and will be referred throughout this study as ICG1, ICG2 and ICG3, where ICG stands for instant coffee grounds. The rest of the samples were products of the retail coffee market for use in Espresso machines and will be referred as RCG1, RCG2 and FRCG, where RCG stands for retail coffee grounds and FRCG for fresh retail coffee grounds. Samples FRCG and RCG2 were provided from the same coffee shop.

### Coffee Ground Moisture Removal and Water Content Determination

Throughout this work, the term moisture content refers to the amount of liquid component of the SCG samples that could be removed thermally, and is not intended as an absolute measure of the presence of elemental H_2_O. Moisture content determination and complete (or partial) moisture removal for subsequent oil extraction was accomplished through thermal drying. Different sample thicknesses ranging between 7.5 and 15 mm were used, while oven temperatures of 100–200 °C were selected. For determination of the total moisture content, oven drying was conducted until there was no change in the measured weight between subsequent measurement intervals. Similarly with the study conducted by Gómez-de la Cruz et al. who investigated the drying of SCGs, any loss of volatiles due to thermal drying was not appreciated when the temperature applied was equal or lower than 200 °C [9].

For all samples, complete moisture removal was achieved after an oven drying period of between 5.5 and 6 h. Equation 1 gives the moisture content value on a weight basis:

1 $$\% ~M=\frac{{{W_1} - {W_2}}}{{{W_1}}}~ \times 100$$

where M, W_1_ and W_2_ are the moisture content on a mass basis, initial coffee weight and final coffee weight after drying respectively.

Partially wet SCGs were obtained by drying SCGs for a shorter duration than that required for complete moisture removal, with the desired water content predicted based on previously experimentally determined moisture removal rates for specific coffee masses, sample type and drying conditions. Equation 2 yields the moisture content of coffee sample after the drying process:

2 $$\% ~M=\frac{{{W_2} - {W_{exp}}}}{{{W_1}}}~ \times 100$$

where M, W_2,_ W_exp_, W_1_ are the moisture content, weight of the sample after drying, expected weight of sample if complete drying was performed, and initial weight of the sample respectively. Equation 3 defines the value of W_exp_:

3 $${W_{exp}}={W_1} - ({W_1} \times ~{M_{average}}\% )$$

where M_average_ is the average experimentally determined moisture content of a specific sample of coffee grounds.

### Particle Sizing of Dry SCGs

Particle sizing was undertaken using test sieves with pore sizes of 1700, 850, 500, 425, 355, 300, 150, 89 and 75 μm. The sieves were shaken at a speed of 40 rpm for 10 min. Equation 4 gives the percentage of SCG particles of a specific diameter range on a mass per mass basis:

4 $$\% ~P=\frac{{{W_2} - {W_1}}}{{{W_{total}}}}~ \times 100$$

where P, W_1_, W_2_ and W_total_ are the percentage of certain size particles, initial weight of sieve, weight of sieve after shaking and total weight of coffee used respectively. An average particle diameter for each coffee sample used was also determined. Equation 5 shows the calculation of the average particle diameter:

5 $$D=\frac{{\mathop \sum \nolimits_{{i=1}}^{n} \left[ {\frac{{\left( {S~ \times P} \right)}}{{100}}} \right]}}{n}$$

where D, S, P and n are the average particle diameter size, sieve size, percentage of particles that remain in a specific sieve and number of test sieves used respectively.

### Solvent Extraction (Soxhlet Method)

Oil extraction from dry or partially dry SCGs with *n-*hexane was undertaken through the Soxhlet method [28]. A 250 ml Soxhlet apparatus was used in conjunction with a high purity glass microfiber thimble of 30 mm diameter and 100 mm height. For each extraction, 22.5 g of SCGs were placed in the thimble and a solvent quantity of between 100 and 200 ml was used.

Following an extraction, lipids remain dissolved in the solvent solution and rotary evaporation was used to rapidly remove excess solvent by applying heat to a rotating round bottomed flask at a reduced pressure. Any remaining traces of solvent were removed by nitrogen-assisted evaporation or thermal drying. The oil yields achieved were calculated following solvent separation procedures as per Eq. 6.

6 $$\% ~oil~recovered=\frac{{{W_2} - {W_1}}}{{{W_3}}}~ \times 100$$

where W_1_ is the weight of the empty glass vial, W_2_ is the weight of the vial plus the extracted oil and W_3_ is the weight of the dry SCGs.

### Pilot Plant Solvent Extraction

**Fig. 1:**
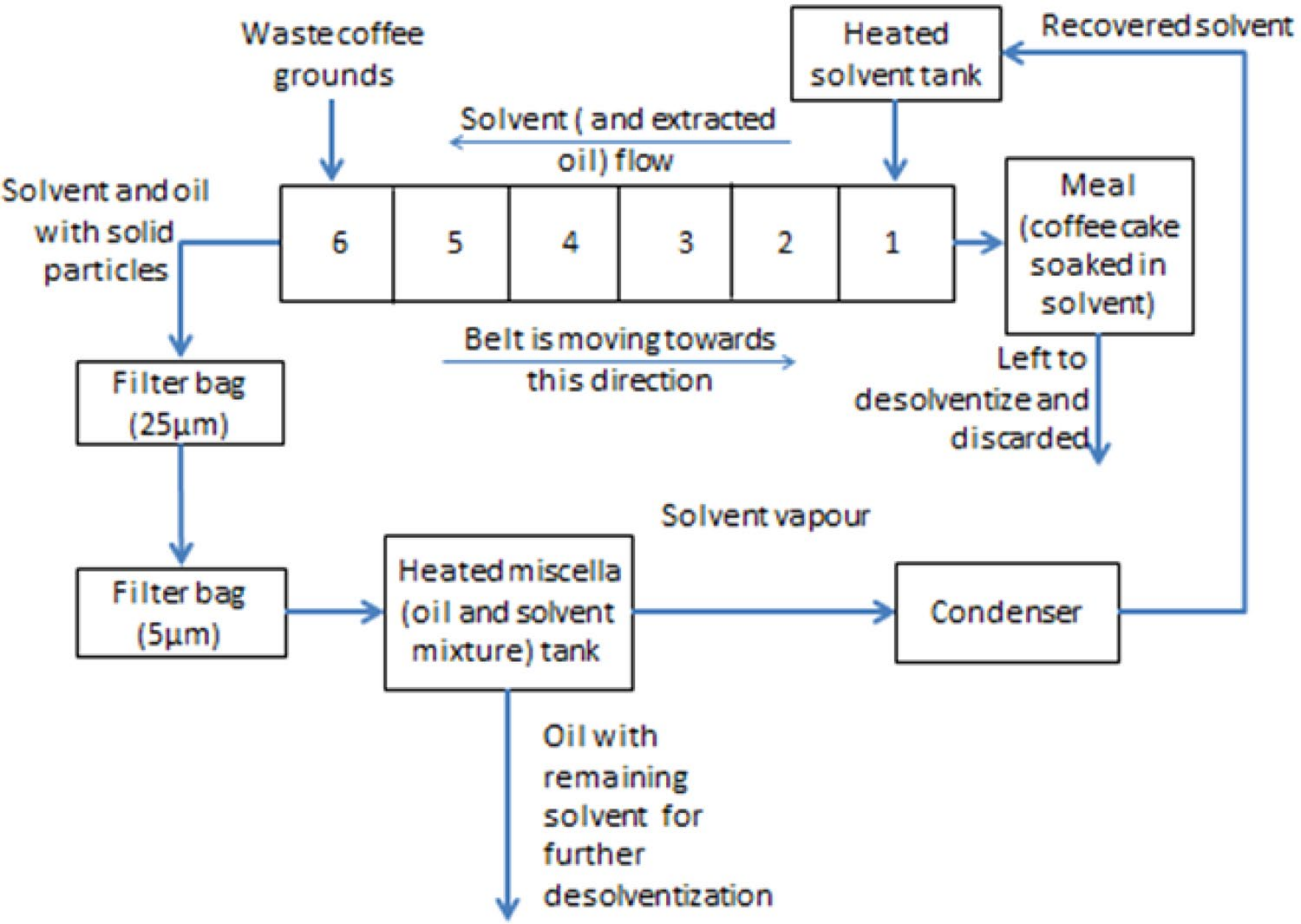
Overall schematic of the pilot plant used in extraction experiments

Large scale solvent extraction experiments were conducted by a tolling manufacturer (TM) in a pilot plant scale batch counterflow solvent extractor of capacity of 8 kg of SCGs per batch. The extractor consisted of six compartments containing a conveyor belt and was heated to 60 °C through a water pipe running beneath the compartments. Figure 1 shows the flow diagram of the TM’s pilot plant.

SCGs were added to compartment 6 (Fig. 1), while hexane was added to compartment 1 at a flow rate of 380 ml/min, resulting in countercurrent contact of feedstock and solvent. The process was performed at a vacuum of 770 mbar so as to reduce the boiling point of the solvent. Extraction experiments with this plant of durations of 1 and 2 h were conducted with SCG samples containing moisture contents of 5 and 10% w/w.

Subsequent to leaving compartment 6 (Fig. 1), the meal (SCGs soaked in solvent) and the miscella (oil and solvent mixture) were collected in separate tanks. The oil and solvent mixture was filtered (to remove any solid particles) twice through bag filters with pore sizes of 25 and 5 μm before reaching the collection tank. This tank was subsequently heated and the resulting solvent vapour was conveyed to a condenser for recovery. The resulting oil, with remaining solvent traces, was subjected to rotary evaporation for further refining of the oil. The oil yield of the process was calculated according to the method described in “Solvent Extraction (Soxhlet Method)”.

### Fatty Acid Profile and FFA Content Determination

The FA composition of the lipids extracted at the TM’s pilot plant was determined by gas chromatography (GC) coupled with a flame ionization detector, following transesterification of the oil sample with methanol in the presence of sulphuric acid at 60 °C to yield FAMEs. The GC was equipped with an Agilent Capillary column CP-Wax 52 CB FS, the injector temperature set to 230 °C and the detector temperature set to 300 °C. The carrier gas was nitrogen at a flow rate of 0.8 ml/min, with the oven temperature initially kept at 170 °C for 3 min and then heated at a rate of 4 °C/min up to 220 °C. Quantitative analysis was carried out using standard FAMEs as internal standard. The FFA content of the oil samples from both the pilot plant and the laboratory scale solvent extractions prior to transesterification was determined through a method of titration with phenolphthalein as the indicator [39].

## Results and Discussion

### Feedstock Characterization

The different coffee samples used in this study were characterized in terms of moisture content, average dry particle diameter and maximum oil yield recovered by Soxhlet on dry weight basis according to the methods described in “Coffee Ground Moisture Removal and Water Content Determination”, “Particle Sizing of Dry SCGs”, “Solvent Extraction (Soxhlet Method)” respectively with the results presented in Table 2. The standard deviations calculated from three experimental repeats are also presented, while the oil contents were achieved with a coffee to solvent ratio of 1:9 w/v after 8 h of extraction.

**Table 2:** Feedstock characterization in terms of moisture and oil content and average particle diameter

Sample	Moisture content (%) w/w	Oil content on dry weight basis (%) w/w	Average particle diameter (mm)
ICG1	57.4 ± 1.0	24.26 ± 1.62	0.66 ± 0.02
ICG2	69.9 ± 0.9	30.45 ± 0.94	1.18 ± 0.06
ICG3	63.3 ± 2.4	25.84 ± 1.21	0.83 ± 0.03
RCG1	64.2 ± 0.6	14.84 ± 0.85	0.76 ± 0.08
RCG2	54.7 ± 1.3	13.38 ± 0.83	0.58 ± 0.03
FRCG	3.3 ± 0.4	12.33 ± 1.10	0.50 ± 0.01

The measured SCG moisture contents are similar to those reported by Kondamudi et al. (50–60% w/w), Oliveira et al. (>50% w/w), Haile (57.6% w/w), Gomez-de la Cruz et al. (58.5% w/w) and Abdullah and Bulent Koc (67% w/w) and higher than the moisture content found by Deligiannis et al. [10] (18–45% w/w) and Ahangari and Sargolzaei (48% w/w) [6, 7, 9, 10, 13, 16, 26]. The variance of the moisture results can be likely attributed to the different coffee brewing procedures that significantly increase the moisture content of SCGs, however there is not a clear difference between ICG and RCG samples (Table 2). Furthermore, the samples have been stored and transported in bulk, something that could have potentially affected their moisture content. The moisture content of the FRCG sample is significantly lower relative to the water residing in other samples, as it has not been used for coffee brewing and therefore has not been treated with water. The water present in this sample can therefore be attributed to long storage and the hydroscopic nature of the coffee grounds [40].

Table 2 shows that SCGs from the instant coffee industry contain significantly more oil than retail SCGs. This is in agreement with the range of oil yields shown in Table 1 which were obtained from retail SCGs and contain amounts of lipids in similar ranges to those of the RCG samples of the present study [7, 8, 10, 12, 13, 16, 17]. The oil contents of FRCG and RCG2 samples are quite similar suggesting that the coffee brewing procedure used, an Espresso machine, does not remove lipids. This is in contrast with the study of Jenkins et al. who extracted more oil from fresh coffee grounds (11–14% w/w) relative to SCGs (7–13% w/w) [11]. Other studies have suggested that different brewing procedures of the fresh coffee could lead to variation in the concentration of other substances in the SCGs, thus potentially altering the obtained crude oil yield [8].

The determination of the average particle diameter of the SCG samples used showed that there is a correlation between increasing particle diameter and increasing moisture content for both the instant and retail coffee groups. Plotting the two variables for all SCG samples revealed a linear relationship giving the regression equation y = 24.2x + 42.4 with a coefficient of determination (R^2^) value of 0.88 which is a statistical measure of how close the data are to the fitted regression line, while ICG samples gave the equation y = 23.3x + 42.8 with R^2^ = 0.97. This can likely be attributed to larger interstitial gaps forming between particles of larger diameter that possibly retain more unbound moisture. The particle size of SCGs can also impact on drying rate as it modifies the distance the bound water has to diffuse [41]. The average particle diameter of FRCG increases after brewing and the larger size of RCG2 particles can be explained by the compression and compaction of coffee grounds during the brewing process, leading some particles to compact together. The low variance of FRCG particle diameter can likely be attributed to the coffee machine grinder that reduces the roasted coffee beans to a predetermined size. Any correlation between particle diameter and oil recovery is investigated in “Impact of Soxhlet Process Factors on Oil Yield”.

### Moisture Removal From SCGs

**Fig. 2:**
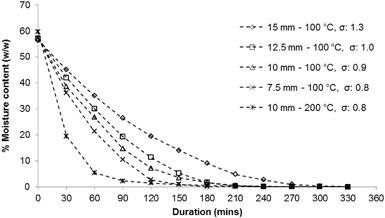
Moisture removal over time with varying sample thickness and drying temperature

Drying experiments were conducted with ICG1 samples of various thicknesses at temperatures of 100 and 200 °C so as to investigate the effect of sample thickness and drying temperature on the moisture removal efficiency. Figure 2 shows the relationship between moisture content and duration of drying, while the standard deviations (σ) were calculated after three sets of experiments with each sample and represent the reproducibility of the obtained results.

Figure 2 shows that a decrease of the sample thickness leads to higher rates of moisture removal and a significant decrease of the time required to remove the bulk of the residing water. Furthermore, drying at 200 °C considerably accelerates the process of removing water from SCCs relative to a drying temperature of 100 °C, when samples with the same thickness are used. In particular, 90% of the total moisture present was removed after 60 min of drying at 200 °C, while approximately 150 min was required for the same level of water reduction at 100 °C. Therefore, increasing the drying temperature and reducing the sample thickness can decrease the duration required for moisture removal from SCGs, an observation which is in agreement with the study conducted by Gomez-de la Cruz et al. [9].

**Fig. 3:**
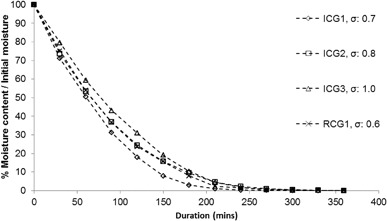
Moisture removal over time with different coffee samples

Figure 3 shows the percentage of moisture relative to the initial moisture content over drying time at 100 °C for samples ICG1, ICG2, ICG3 and RCG1 with a thickness of 10 mm. Three experimental repeats were performed with each sample in order to calculate the standard deviation (σ) of the mean for each point.

It can be seen in Fig. 3 that all samples show similar drying rates, with moisture content approaching zero after 4 h. It is interesting to note that the required drying time for complete water removal is not significantly affected by the different initial moisture contents of the samples or mean particle size (Table 2).

### Impact of Soxhlet Process Factors on Oil Yield

**Fig. 4:**
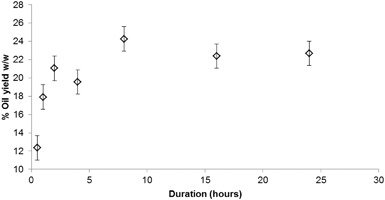
Oil yields per mass of dry coffee weight achieved at different Soxhlet extraction durations

This section investigates the effect of extraction parameters including duration, coffee to solvent ratio, moisture content and particle size on the oil yield obtained from the various SCG samples. Figure 4 shows the extracted oil yields from ICG1 on a dry weight basis with varying Soxhlet duration and a constant dry coffee ground to solvent ratio of 1:9 w/v. The error bars correspond to the standard deviation calculated from 21 experimental repeats in the range of 0.5 and 24 h of extraction duration and represent the reproducibility of the obtained oil yield percentages.

Figure 4 shows that the highest oil yield was achieved at 8 h of solvent extraction, with a subsequent slight decrease of oil yields obtained at durations greater than 8 h. This is in agreement with Picard et al. and Sayyar et al. who investigated oil extraction via Soxhlet from roasted Robusta grounds and jatropha seeds respectively and found a duration of 8 h to be ideal for oil recovery [29, 32]. Figure 4 also shows that the extracted oil yield reduces considerably when the duration of Soxhlet extraction is <2 h. This is likely because the short duration of extraction does not allow sufficient time for the recirculating solvent to extract the total available oil from the SCGs, and is in agreement with previous studies investigating the extraction of lipids from other oilseeds through Soxhlet which suggest that durations longer than 3.5 h result in more efficient extractions [30, 31].

The effect of the dry coffee (ICG1) to solvent ratio on the oil yield was examined at a constant Soxhlet duration of 8 h with hexane. The coffee to solvent ratio ranged between 1:9 and 1:4 w/v and this caused a variation in the obtained oil yield between approximately 24.6 ± 1.33 and 17.6 ± 0.96% w/w with a tendency for higher yield in the lower coffee to solvent ratios. The same correlation between decreasing seed to solvent ratio and increasing oil yield was observed from Pichai and Krit and Sayyar et al. at oil extraction from SCGs and jatropha seeds respectively [15, 32].

**Fig. 5:**
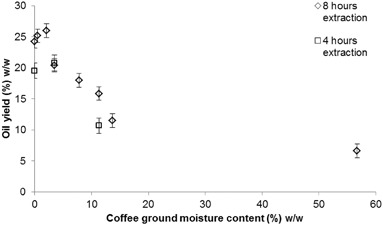
Oil yields on a dry weight basis versus the respective moisture content for 4 and 8 h extractions

The effect of various ICG1 moisture levels on the oil yield was examined with hexane used at a coffee to solvent ratio of 1:9 w/v, while three repeats were performed for every experiment. Figure 5 shows the relationship found between moisture content and achieved oil yield on a dry weight basis, while the error bars represent the standard deviation of the mean for each point.

Figure 5 shows that a moisture content of approximately 2% w/w does not affect the process negatively and actually appears to be beneficial as it results in a slightly higher amount of oil extracted than at lower moisture content. Moisture contents greater than 2% w/w resulted in lower oil yields, however a SCG sample with moisture level of roughly 3.5% w/w yields ~20% w/w oil at both the durations of 4 and 8 h, which is not a significant drop from the peak yield of ~25% w/w. These results are inconsistent with previous studies that suggested a moisture content of 9–11% w/w as ideal for Soxhlet oil extraction, suggesting a difference of optimum extraction conditions between SCGs and other oilseeds like soybeans [30, 31, 34, 35].

**Fig. 6:**
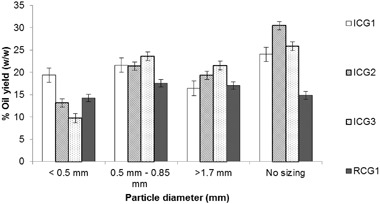
Oil yield of various SCG samples when different particle sizes are used

The oil yield extracted from coffee particles of various size fractions was also evaluated. Dry ICG1, ICG2, ICG3 and RCG1 samples were used at a fixed coffee to hexane ratio of 1:9 w/v, and extraction duration of 8 h. Each of the SCG samples was split into fractions of the following particle diameter: < 0.5 mm, 0.5 mm < d < 0.85 mm and d > 1.7 mm with the yield from each sample without splitting by size also shown for reference (the average particle diameter of the different SCG samples can be found in Table 2). Figure 6 presents the oil yield achieved from different particle size fractions of each coffee sample, while the error bars represent the standard deviation of the mean calculated from three repeats.

Figure 6 shows that when sizing has been conducted, an intermediate particle diameter between 0.5 and 0.85 mm leads to higher oil recoveries from all the SCG samples. This is in agreement with Sayyar et al. who achieved the highest oil recoveries from jatropha seeds with particle size meal between 0.5 and 0.75 mm [32]. With the exception of ICG1, larger particles result in higher oil recoveries than smaller ones, something that can possibly be attributed to the compaction and agglomeration of the fine particles that result in reduction of the surface area. This is in contrast with previous studies on the solvent extraction of lipids from oilseeds which suggested that a small seed particle size of 0.2–0.3 mm is beneficial for the extraction efficiency of the process [30, 31, 35–38]. Finally, for all coffee samples, except RCG1, higher oil yields were obtained without sizing, suggesting that a mix of different size particles facilitates the solvent and oil flow, therefore leading to higher oil recoveries.

### Oil Yield Achieved at a Large Scale Solvent Extraction

Solvent extraction experiments at pilot plant scale were undertaken to validate trends observed in the laboratory scale experiments, however, the only variables that could be modified were the duration of the process and the moisture content of the SCGs. As completely dry samples of the required mass were not available, ICG1 samples with moisture contents of 5 and 10% w/w were utilized. At extraction durations of 1 and 2 h with 5% w/w moisture content, oil yields of 17.3 and 20.8% w/w were obtained respectively. These oil yields are directly comparable with those achieved at the same durations with the Soxhlet method (17.9 and 21.06% w/w) and dry ICG1 samples (Fig. 5) and support the finding that increasing duration (below 8 h) increases extracted oil yields.

The oil yield achieved from an ICG1 sample with 10% w/w moisture after 1 h of extraction was 19.3% and this suggests that an intermediate portion of moisture in the feedstock does not hinder the large scale extraction process. The presence of water might in fact be responsible for the extraction of additional water soluble compounds such as proteins, phosphatides and carbohydrates, increasing the overall extraction yield [35, 42]. This behaviour comes in contrast with the results obtained through Soxhlet extraction when samples of various moisture contents were used and suggests that the pilot plant is less sensitive to the presence of water, potentially due to the countercurrent contact of feedstock and solvent.

Complete solvent removal could not be achieved from the pilot plant extracted oil sample (due to the limitation of equipment available onsite) and the actual oil yields may therefore be somewhat lower than the measured values. Notwithstanding the limitation of the results, considering the oil yield of ICG1 sample (24.26% w/w) obtained at the laboratory scale, the results of pilot plant scale extraction process showed the laboratory scale Soxhlet experiments to be representative of industrial scale processes that could be utilised for extraction of lipids from SCGs.

### Fatty Acid Profile

The determination of the FA profile of the ICG1 oil samples extracted at the pilot plant was carried out according to the method described in “Fatty Acid Profile and FFA Content Determination”. Table 3 shows the FA composition of the oil samples, along with FA compositions of SCG oils extracted though solvent extraction in previous studies. Table 3 also includes the FA profiles of soybean and palm oil, two major biodiesel feedstocks [7], for comparison purposes. Linoleic (C18:2), palmitic (C16:0), oleic (C18:1), stearic (C18:0), eicosanoic (C20:0) and linolenic (C18:3) were the FAs with the highest weight percentages in a decreasing order of magnitude.

**Table 3:** FA profile of examined ICG1 oil samples and other selected oil compositions from literature

	C12:0	C14:0	C16:0	C18:0	C18:1	C18:2	C18:3	C20:0	SFA	UFA	PUFA
ICG1, 5% moisture, 60 min	ND	TR	32.4	8.1	10.2	41.7	1.1	3.8	45.4	53.9	42.9
ICG1, 5% moisture, 120 min	ND	ND	34.6	8.1	9.3	40.7	1	4	47.8	52.2	41.8
ICG1, 10% moisture, 60 min	ND	TR	32.4	7.5	10.1	43.1	0.9	3.1	44.1	55.3	44.1
Other studies of coffee lipids											
[13]	3.54	1.97	43.61	6.58	8.15	32.41	1.3	2.44	58.14	41.86	33.71
[12]	3.57	1.99	43.65	6.49	8.15	32.45	1.31	2.39	58.09	41.91	33.76
[6]	NM	NM	35.8	8.1	13.9	37.3	NM	3.2	47.1	51.2	37.3
[43]	ND	ND	32.8	7.1	10.3	44.2	1.5	2.6	42.5	56	44.2
Other vegetable oils											
Palm oil [44]	NM	NM	44	4	40	10	NM	NM	48	50	10
Soybean oil [45]	NM	TR	9	4	28	49.5	NM	NM	13	77.5	49.5

*ND* not detected, *NM* not mentioned, *TR* traces, *SFA* saturated fatty acids, *UFA* unsaturated fatty acids, *PUFA* polyunsaturated fatty acids

Table 3 shows that coffee oil extracted from 5 and 10% w/w coffee samples consisted of both saturated (44.1–47.8% w/w) and unsaturated FAs (52.2–55.3% w/w), with the portion of polyunsaturated FAs ranging from 41.8 to 44.1% w/w. The bulk of the extracted oils (92.4–93.1) is comprised of linoleic, palmitic, oleic and stearic acid, followed by small amounts of eicosanoic and linolenic, while other FAs that are not disclosed in the table were found in considerably lower amounts (<0.5). The absence of significant differences in the FA profiles of the three examined oil samples suggests that the moisture content of SCGs and the duration of solvent extraction do not have an important effect on the extracted oil composition.

These results are in good agreement with SCG oil FA profiles obtained from previous studies [6, 11, 43, 46]. Very similar FA profiles were found by Jenkins et al., who demonstrated that the majority of examined coffee oils from fresh and waste samples have similar composition irrespective of the origin, type of bean and brewing process [11]. Furthermore, Martin et al. and Ratnayake et al. found that no significant differences could be detected in the FA composition of the oil from green and roasted beans [46, 47]. In particular, the roasting process only increases the trans FA levels (C18:2ct and C18:2tc) [23]. However, experiments performed by Couto et al., Ahangari and Sargolzaei and Kondamudi et al. revealed slightly different SCG oil compositions and suggest that differences can occur [7, 12, 13].

The main differences are related to the percentages of linoleic and palmitic acid, which were higher and lower than those measured in the present study respectively (Table 3). Martin et al., has also noticed that the SCG oils can be categorized into two classes based on their FA composition: those with low palmitic (<40%) and high linoleic (>40%) acids and reversely, those with high palmitic (>40%) and low linoleic (<40%) acids [46]. Such differences in composition can be most likely attributed to the origin of the coffee sample and the different blends of coffee varieties that may have been used [46]. For example, Jenkins et al. found that the oil composition of Vietnamese coffee was very different from other samples tested [11].

It can be seen from Table 3 that coffee derived oil shares the major FAs in somewhat similar percentages with soybean oil and has an almost identical saturated to unsaturated FA ratio as palm oil. Furthermore, SCG oil obtained by Haile, which has a very similar FA profile to oil extracted from ICG1, resulted in biodiesel that was found to be within the standard limits (EN 14214) for parameters including density, kinematic viscosity, iodine value, AV and flash point [6]. The suitability of an oil as a biodiesel feedstock is highly dependent on the FA profile [48], and the relatively high percentage of oleic acid in the coffee oil (9.3–10.2% w/w in oil extracted from ICG1—Table 3) is potentially beneficial for biodiesel production, as the relatively long alkyl chain length with only one double bond results in high oxidative stability and a low melting point [13, 49]. On the contrary, the high level of polyunsaturated fatty acids in the obtained coffee oil is a disadvantage for biodiesel production as it is closely related to the oxidation rate and the degradation tendency of the fuel [49–51]. One feasible solution to this issue would be to treat the resulting biodiesel with oxidation inhibitors [51, 52]. In addition, higher degrees of alkyl moiety unsaturation have been implicated in higher engine exhaust NOx emissions, however, this has found to be secondary to properties such as fuel cetane number [53].

### FFAs in the Coffee Oil

**Fig. 7:**
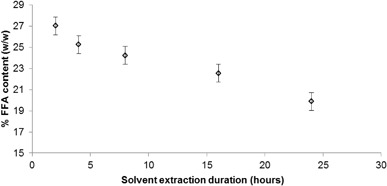
Correlation of the % FFA content of extracted coffee oil with the duration of solvent extraction

The acid value and FFA content of a range of the extracted oil samples were measured according to the method described in “Fatty Acid Profile and FFA Content Determination”. Figure 7 shows the FFA content of ICG1 oil samples that were obtained at different durations of Soxhlet extraction with hexane and at a coffee to solvent ratio of 1:9 w/v. The error bars present show the standard deviation calculated from 15 experimental repeats of the FFA content determination with oils extracted at Soxhlet durations ranging from 2 to 24 h.

Figure 7 shows that the FFA content of the extracted oil decreases with increasing duration of solvent extraction. It is suggested that FFAs, as smaller molecules than triglycerides or diglycerides are the first to be extracted through solvent extraction and consequently the fraction of these is higher when the duration of extraction is short. However, as the duration of extraction increases, more triglycerides are recovered and therefore the percentage of FFAs in the collected oil decreases. Similar results were seen in the FFA content of oils extracted at the pilot plant from ICG1 sample with 5% w/w moisture content, with an extraction duration of 1 h resulting in an oil of FFA content of 33.01% w/w, while an extraction duration of 2 h yielded oil with FFA content of 29.48% w/w.

The FFA content of oil samples extracted from dried samples ICG1, ICG2 and RCG1 after 8 h of extraction with *n-*hexane and a coffee to solvent ratio of 1:9 w/v was found to be 24.24, 38.3 and 21.61% w/w respectively. A comparison between the oil content of samples ICG1, ICG2 and RCG1 (Table 2) and the respective FFA content of the oil extracted from those samples reveals a weak correlation between increasing oil content and FFA content.

Such FFA levels are relatively high when compared with levels of FFAs found in previous studies, which commonly ranged from ~3 to ~20% when hexane was the solvent used [6, 8, 27, 54–56], while values as low as 0.31 [10] and as high as 59% [17] have been reported. Since the same extraction method and solvent has been used in all these studies, this variation further suggests that there is significant variation in the physical properties of different SCG samples. The duration of storage of the coffee samples could be one explanation for high FFA contents as Speer and Kolling-Speer, found that prolonged storage of green coffee (18 months), especially at conditions of relatively high storage temperature (25–40 °C) and moisture content (11.8%), led to an increased concentration of FFAs in the oil [23].

## Conclusions

1. The apparent moisture content of various SCG samples was found to range between 54 and 70% w/w, and the drying of SCGs was accelerated by decreases in cake thickness and increasing drying temperature. A drying temperature of 200 °C was found to more quickly reduce the SCG moisture content to 2% w/w relative to a temperature of 100 °C, however, a constant duration of 5 h drying was required for complete moisture removal at both temperatures.
2. A relationship between SCG initial moisture content and particle size distribution was observed, samples containing particles of large average diameter tending to possess higher moisture content. Soxhlet extraction experiments with coffee samples of discrete particle diameters found a mix of particles of different diameters to lead to higher oil yield.
3. The oil content of the different SCG samples ranged from 13.4 to 30.4% w/w, with SCGs from the instant coffee industry containing significantly more oil than retail SGCs, while extraction from the same sample of coffee grounds before and after brewing in Espresso machine was found to have no significant impact on the obtained oil yield.
4. An extraction duration of 8 h was found to result in the highest oil yields by Soxhlet extraction, while shorter durations resulted in reduced but potentially acceptable oil yield. A similar result was observed during pilot scale solvent extraction, with an increase of extraction duration from 1 to 2 h increasing oil yields.
5. Soxhlet experiments with partially dried SCGs showed that minimal moisture content results in slight oil yield increase relative to completely dry SCGs. Experiments in the pilot plant revealed a reduced sensitivity to water relative to Soxhlet extraction, as high yields were obtained when samples with moisture content of 10% were used.
6. The FA profile of the extracted oils mostly consisted of linoleic, palmitic, oleic and stearic acid. Similarity of the oil FA profile to that of other vegetable oils commonly used as biodiesel feedstocks, as well as to SCG derived biodiesel from a previous study that was found to be within the standard limits [6], suggests that the obtained oil is suitable for biodiesel production. The FFA content of the oils extracted increased with decreasing duration of extraction, while in the case of various SCG ground samples, FFA content was found to increase as a percentage of the extracted oil as the total oil yield increased.

## Abbreviations

SCG: Spent coffee ground
FFA: Free fatty acid
FA: Fatty acid
FAME: Fatty acid methyl ester
TM: Tolling manufacturer
